# Supplementary material for: Metagenomics uncovers dietary adaptations for chitin digestion in the gut microbiota of convergent myrmecophagous mammals
Source: mSystems. 2023 Aug 31;8(5):e00388-23. doi: 10.1128/msystems.00388-23 (PMC10654083; doi:10.1128/msystems.00388-23)
Supplement: Fig. S1 — Phylogeny of the 314 high-quality selected bins reconstructed from long- and short-read assemblies. [file msystems.00388-23-s0001.pdf]

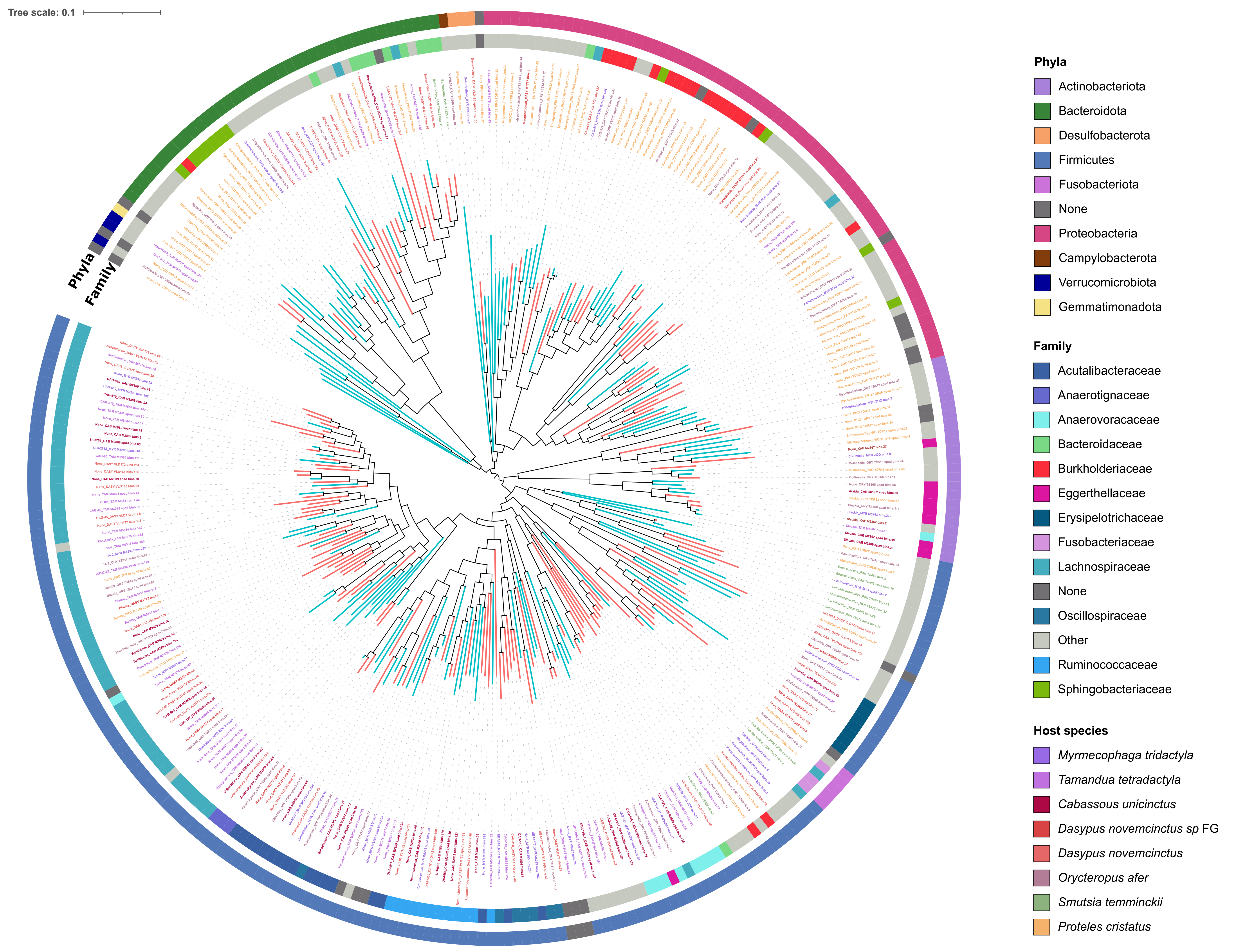

Fig S1 Phylogeny of the 314 high-quality selected bins reconstructed from long read assemblies (n = 156; red branches) **and short-read assemblies** (n = 158; blue branches)**.** Circles respectively indicate (from inner to outer circles): the bacterial family and phyla the bin was assigned to based on the Genome Taxonomy Database release 7 (38). Colored sequence names indicate the host species. Bins' names of the myrmecophagous-specific clades are indicated at leaves of the phylogenetic tree together with the genus to which they were assigned to.
