# Supplementary material for: Metagenomics uncovers dietary adaptations for chitin digestion in the gut microbiota of convergent myrmecophagous mammals
Source: mSystems. 2023 Aug 31;8(5):e00388-23. doi: 10.1128/msystems.00388-23 (PMC10654083; doi:10.1128/msystems.00388-23)
Supplement: Fig. S2 — Myrmecophagous-specific clades within Bacteroidetes and Proteobacteria. [file msystems.00388-23-s0002.pdf]

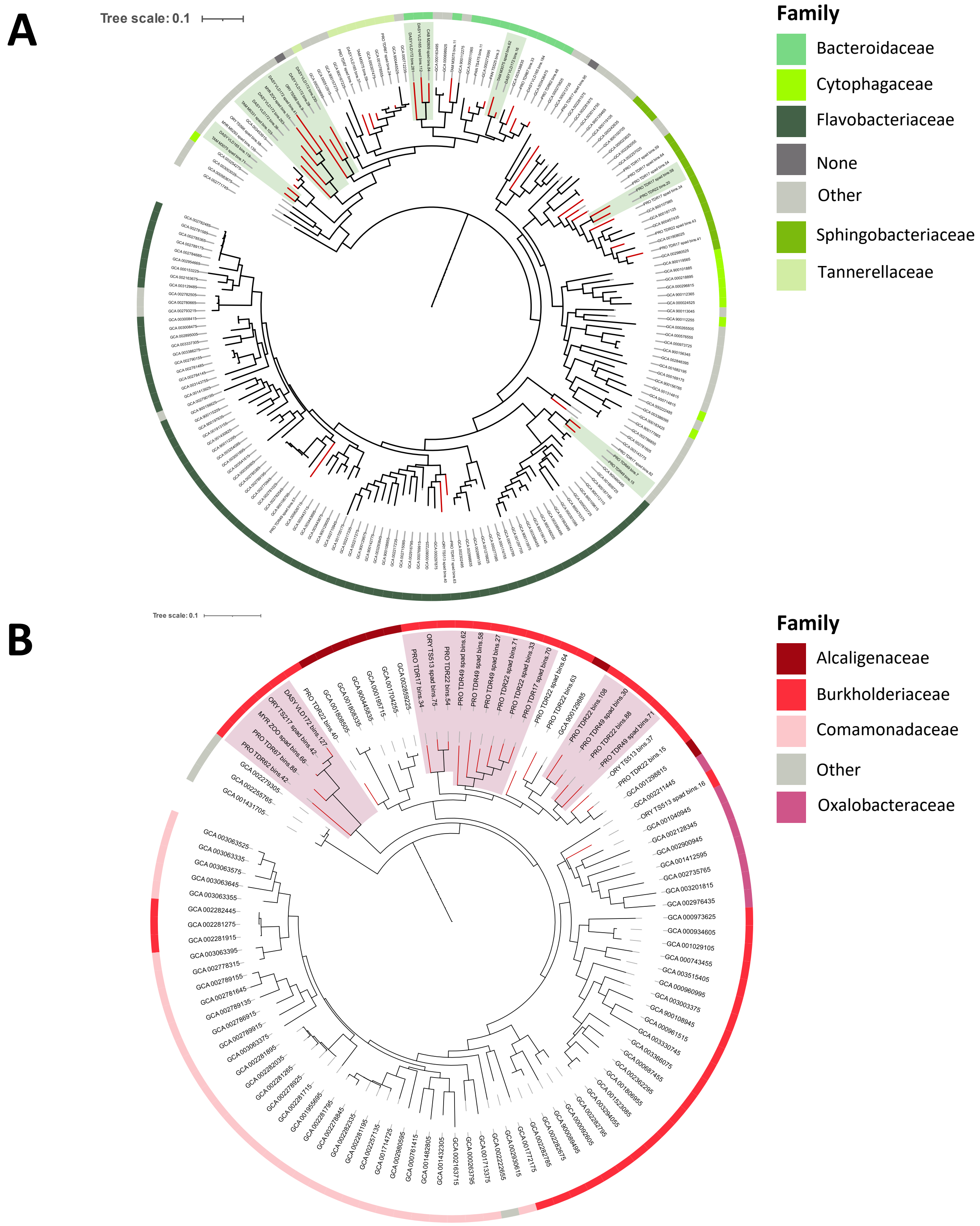

Fig S2 Myrmecophagous-specific clades within Bacteroidetes (A; green highlights) and **Proteobacteria** (B; pink highlights). The two trees are subtrees of Fig. 1. Outer circles indicate the bacterial family to which these genome bins were assigned based on the Genome Taxonomy Database release 7 (38).
