# Supplementary material for: Metagenomics uncovers dietary adaptations for chitin digestion in the gut microbiota of convergent myrmecophagous mammals
Source: mSystems. 2023 Aug 31;8(5):e00388-23. doi: 10.1128/msystems.00388-23 (PMC10654083; doi:10.1128/msystems.00388-23)
Supplement: Table S3 — Proportion of chitinolytic genome bins (genomes having at least one GH18 with an active chitinolytic site) detected in the nine focal myrmecophagous species. [file msystems.00388-23-s0007.pdf]

Table S3 Proportion of chitinolytic genome bins (genomes having at least one GH18 with an active chitinolytic site) **detected in the nine focal myrmecophagous species.**

Species	# chitinolytic bins detected	total # detected bins	# chitinolytic bins / total # bins detected
Cabassous unicinctus	46	113	0.407079646
***Dasypus sp. nov.* FG**	48	105	0.457142857
Dasypus novemcinctus	50	129	0.387596899
Dasypus kappleri	2	25	0.08
Myrmecophaga tridactyla	48	141	0.340425532
Orycteropus afer	15	97	0.154639175
Smutsia temminckii	5	18	0.277777778
Proteles cristatus	12	104	0.115384615
Tamandua tetradactyla	37	121	0.305785124
