# Supplementary material for: Metagenomics uncovers dietary adaptations for chitin digestion in the gut microbiota of convergent myrmecophagous mammals
Source: mSystems. 2023 Aug 31;8(5):e00388-23. doi: 10.1128/msystems.00388-23 (PMC10654083; doi:10.1128/msystems.00388-23)
Supplement: Table S4 — Detailed sample information for the eight soil samples collected in South Africa. [file msystems.00388-23-s0008.pdf]

Table S4 Detailed sample information for the eight soil samples collected in South Africa.

Sample	Sample type	Species	Common name	Class	Order	Family	Location
**TDR012**	Soil from Midden #1	*Proteles cristatus*	Southern ardwolf	Mammalia	Carnivora	Hyaenidae	Tussen Die Riviere Reserve
**TDR014**	Nest fragments and soil	*Trinervitermes sp.*	Snouted termites	Insecta	Blattodea	Termitidae	Tussen Die Riviere Reserve
**TDR019**	Soil from Midden #2	*Proteles cristatus*	Southern ardwolf	Mammalia	Carnivora	Hyaenidae	Tussen Die Riviere Reserve
**TDR023**	Soil from Midden #3	*Proteles cristatus*	Southern ardwolf	Mammalia	Carnivora	Hyaenidae	Tussen Die Riviere Reserve
**TS218**	Soil from midden	*Orycteropus afer*	Aardvark	Mammalia	Tubulidentata	Orycteropodidae	Tswalu Kalahari Reserve
**TS274**	Nest fragments and soil	*Trinervitermes sp.*	Snouted termites	Insecta	Blattodea	Termitidae	Tswalu Kalahari Reserve
**TS281**	Soil from midden	*Orycteropus afer*	Aardvark	Mammalia	Tubulidentata	Orycteropodidae	Tswalu Kalahari Reserve
**TS298**	Soil from midden	*Orycteropus afer*	Aardvark	Mammalia	Tubulidentata	Orycteropodidae	Tswalu Kalahari Reserve
